# iNOS/NO is required for IRF1 activation in response to liver ischemia-reperfusion in mice

**DOI:** 10.1186/s10020-020-00182-2

**Published:** 2020-06-09

**Authors:** Qiang Du, Jing Luo, Mu-Qing Yang, Quan Liu, Caroline Heres, Yi-He Yan, Donna Stolz, David A. Geller

**Affiliations:** 1 Thomas E. Starzl Transplant Institute, Department of Surgery, University of Pittsburgh, 3471 Fifth Avenue, Kaufmann Medical Building, Suite 300, Pittsburgh, PA 15213 USA; 2 Department of Surgery, The Second Xiangya Hospital of Central South University, 139 Renmin Middle Road, Changsha, Hunan People’s Republic of China 410011; 3 Department of Surgery, Shanghai Tenth People’s Hospital, Tenth People’s Hospital of Tongji University, 301 Middle Yanchang Road, Shanghai, 200072 People’s Republic of China; 4 Southern University of Science and Technology, School of Medicine, 1088 Xueyuan Blvd. , Nanshan District, Shenzhen, Guangdong People’s Republic of China 518055; 5 Department of Cellular Biology, University of Pittsburgh, Pittsburgh, PA 15213 USA

**Keywords:** Inducible nitric oxide synthase, Interferon regulatory factor-1, Ischemia-reperfusion, Nitric oxide, Histone deacetylase, p53 up-regulated modulator of apoptosis

## Abstract

**Background:**

Ischemia and reperfusion (I/R) induces cytokines, and up-regulates inducible nitric oxide synthase (iNOS), interferon regulatory factor-1(IRF1) and p53 up-regulated modulator of apoptosis (PUMA), which contribute to cell death and tissue injury. However, the mechanisms that I/R induces IRF1-PUMA through iNOS/NO is still unknown.

**Methods:**

Ischemia was induced by occluding structures in the portal triad (hepatic artery, portal vein, and bile duct) to the left and median liver lobes for 60 min, and reperfusion was initiated by removal of the clamp. Induction of iNOS, IRF1 and PUMA in response to I/R were analyzed. I/R induced IRF1 and PUMA expression were compared between iNOS wild-type and iNOS knockout (KO) mice. Human iNOS gene transfected-cells were used to determine iNOS/NO signals targeting IRF1. To test whether HDAC2 was involved in the mediation of iNOS/NO-induced IRF1 transcriptional activities and its target gene (PUMA and p21) expression, NO donors were used in vitro and in vivo.

**Results:**

IRF1 nuclear translocation and PUMA transcription elevation were markedly induced following I/R in the liver of iNOS wild-type mice compared with that in knock-out mice. Furthermore, I/R induced hepatic HDAC2 expression and activation, and decreased H3AcK9 expression in iNOS wild-type mice, but not in the knock-out mice. Mechanistically, over-expression of human iNOS gene increased IRF1 transcriptional activity and PUMA expression, while iNOS inhibitor L-NIL reversed these effects. Cytokine-induced PUMA through IRF1 was p53 dependent. IRF1 and p53 synergistically up-regulated PUMA expression. iNOS/NO-induced HDAC2 mediated histone H3 deacetylation and promoted IRF1 transcriptional activity. Moreover, treating the cells with romidepsin, an HDAC1/2 inhibitor decreased NO-induced IRF1 and PUMA expression.

**Conclusions:**

This study demonstrates a novel mechanism that iNOS/NO is required for IRF1/PUMA signaling through a positive-feedback loop between iNOS and IRF1, in which HDAC2-mediated histone modification is involved to up-regulate IRF1 in response to I/R in mice.

## Introduction

Interferon regulatory factor-1 (IRF1) is a transcription factor up-regulated in response to various stimuli such as cytokines, double stranded RNA and hormones (Kroger et al. 2002). Nuclear translocation of IRF1 results in the induction of endogenous type I interferon (IFN) (Miyamoto et al. 1988), inducible nitric oxide synthase (iNOS, or NOS2) (Kamijo et al. 1994; Martin et al. 1994) and other genes (Taki et al. 1997). Our previous study identified a critical role for IRF1 in regulation of cell death in liver transplant ischemia and reperfusion (I/R) (Ueki et al. 2010). Liver I/R injury (IRI), a major complication of hemorrhagic shock, resection, and transplantation, is a dynamic process that involves the interrelated phases of local ischemic insult and inflammation-mediated reperfusion injury. Cell death fundamentally determines the extent of liver function (Zhai et al. 2013). The p53 up-regulated modulator of apoptosis (PUMA) is Bcl-2 homology 3 (BH3)-only Bcl-2 family protein, a key mediator in apoptosis (Yu et al. 2001; Nakano et al. 2001; Yu and Zhang 2003), necrosis (Chen et al. 2019) and necroptosis (Chen et al. 2018). PUMA expression, transcriptionally regulated by p53 (Nakano et al. 2001; Yu and Zhang 2003), NF-κB (Wu et al. 2007), forkhead box protein O1 (FOXO1) (Hughes et al. 2011), FOXO3a (You et al. 2006), IRF1 (Gao et al. 2010) and others, is a key step in pathogenesis of IRI in intestine and heart (Wu et al. 2007; Toth et al. 2006).

Histone deacetylases (HDACs) play important roles in regulation of gene expression by removing an acetylation at active genes and resetting chromatin modeling (Seto and Yoshida 2014). They are often related to the suppression of gene transcription, however, many studies show that deacetylation of a histone or non-histone protein is required for IFNα induced gene transcription, and inhibition of HDACs reverses the inducible gene expression (Nusinzon and Horvath 2003). The exact requirement for deacetylation differs among promoters, depending on their specific architecture and regulation scenario (Nusinzon and Horvath 2003). In a genome-wide mapping study, the majority of HDACs in the human genome are associated with chromatin at active genes, and only a minor fraction are detected in inactive genes (Wang et al. 2009). HDAC2 positively regulates cytokine-induced iNOS expression and NO production via HDAC2 physically binding with NF-κB p65 (Yu et al. 2002). However, it has been noticed that NO-induced S-nitrosylation of HDAC2 mediates NO-dependent gene transcription in neurons and hepatocytes, as well as in HEK293 cells (Nott et al. 2008; Kornberg et al. 2010; Nott et al. 2013; Rodriguez-Ortigosa et al. 2014).

Cytokine and chemokine inductions are critical responses to I/R, which triggers immune-mediated injury. Hepatic I/R induces cytokine responses, including TNFα, IFNβ/IFNγ, IL-6, IL-1β, and iNOS starting as early 30 min after I/R and lasting for 8 h (Zhai et al. 2013; Datta et al. 2013; Isobe et al. 1999). Our previous study found that these inflammatory cascades lead to cell death in both non-parenchymal cells (NPCs) and hepatocytes (Ueki et al. 2010). Hepatic I/R induces cytokines in NPCs, which stimulate hepatocytes through their receptors for activations of pro-inflammatory genes and cell death signaling pathways included IRF1 (Ueki et al. 2010).

iNOS/NO is involved in the pathogenesis of hepatic IRI, mainly due to regulating pro-inflammatory genes by stimulating TNFα and IFNγ production and inflammatory responses (Datta et al. 2013). The iNOS gene is transcriptionally regulated by IRF1 (Kamijo et al. 1994; Martin et al. 1994), NF-κB (Taylor et al. 1998), signal transducer and activatior-1 (STAT-1) and other transcription factors (Kleinert et al. 2004). Liver I/R injury occurs after iNOS activation in hepatocytes, which can be attenuated in iNOS knockout (iNOS^−/−^) mice (Hamada et al. 2009). NO modulates the gene expression of many inflammatory mediators including PUMA and p21^CIP1/WAF1^ (p21) (Li et al. 2004; Hemish et al. 2003). Although I/R-induced PUMA expression has been established in intestine and heart IRI (Wu et al. 2007; Toth et al. 2006), the mechanisms of I/R-induced IRF1-PUMA through iNOS/NO has not been elucidated. Here, we provide the evidence that iNOS/NO positively regulates IRF1-PUMA pathway and induces hepatocyte death and liver IRI via a positive-feedback loop between IRF1 and iNOS. Moreover, IRF1 transcriptional activity is partially up-regulated by NO-induced HDAC2 activation.

## Materials and methods

### Human and mouse hepatocytes and reagents

Human (primary) hepatocytes were obtained from the National Institutes of Health (NIH) – funded Liver Tissue and Cell Distribution System core at the University of Pittsburgh. The hepatocytes were cultured in Hepatocyte Maintenance Medium (LONZA, Walkersville, MI) with 5% newborn calf serum. The mouse hepatocytes were isolated from normal mice by an in situ collagenase (type IV) (Sigma Aldrich, Natick, MA) perfusion technique, modified as described previously (Tsung et al. 2006). Unless indicated, cells were stimulated with 250 U/mL human or mouse IFNγ (R&D Systems, or Roche Pharmaceuticals). L-NIL (N^6^-(1-Iminoethyl)-L-lysine hydrochloride) (Cayman Chemical, Ann Arbor, MI) and BYK191023 (2-[2-(4-methoxypyridin-2-yl)-ethyl]-3H-imidazo [4,5-b]pyridine) (Santa Cruz Biotechnology, Santa Cruz, CA), Romidepsin (also known as Istodax) (MedChemExpress, Monmouth Junction, NJ), GSNO (S-Nitrosoglutathione) (Sigma Aldrich, Natick, MA), SNAP (S-Nitroso-N-Acetyl-D,L-Penicillamine) (Cayman Chemical, Ann Arbor, MI) were performed according to the manufacture’s protocol.

### Human cell lines

The 293 T cells were obtained from American Type of Culture Collection and cultured as described previously (Du et al. 2009). HCT116PT53^+/+^ and HCT116PT53^−/−^ were kindly provided by Dr. John Yim (City of Hope National Medical Center) with the permission of Dr. Bert Vogelstein (University of John Hopkins) and cultured in McCoy’s 5A medium (Invitrogen Life Technologies) under the conditions as described for the 293 T cells.

### Mice

C57BL/6 male (8–12 weeks of age) were purchased from the Jackson Laboratory (Bar Harbor, ME). iNOS wild-type mice (iNOS^+/+^), C57BL/6NOS*2*^+/+^ and iNOS knockout mice (iNOS^−/−^), C57BL/6NOS2^−/−^ were kindly provided by Dr. Timothy Billiar (Darwiche et al. 2012; MacMicking et al. 1995) or commercially available as B6.129-NOS2^tm1Lau^/J from Jackson Laboratory. Animal Care and Use Committee of the University of Pittsburgh (IACUC) approved the Animal Protocols, which also included the ethical approval of experiments carried out in adherence to the NIH Guidelines for the Use of Laboratory Animals. Animals were raised in plastic cages under specific pathogen-free conditions. Animals were fed a standard diet for mice and had free access to water in an animal facility of the University of Pittsburgh. The study is compliant with all ethical regulations regarding animal care and use.

### Mouse liver warm I/R models

In order to test whether I/R induced IRF1 signaling requires intact iNOS expression, we utilized iNOS wild-type and knockout mice for hepatic I/R injury. Mouse liver warm I/R procedures were previously described (Tsung et al. 2006). Briefly, a nonlethal model of segmental (70%) hepatic warm ischemia was used. For the I/R protocol, structures in the portal triad (hepatic artery, portal vein, and bile duct) to the left and median liver lobes were occluded with a microvascular clamp (Fine Science Tools, San Francisco, CA) for 60 min, and reperfusion was initiated by clamp removal. Naïve animals underwent anesthesia. Animals were sacrificed at predetermined time points (6, 12, 24 and 48 h) after reperfusion for serum and liver samples.

### PCR

Total RNAs from cells or tissues were isolated with TRIzol reagent (Invitrogen, Carlsbad, CA) and reversely transcribed into cDNA using Sprint RT Complete Products kit (Clontech, Mountain View, CA). Differences in expression were calculated using the Ct method. Quantitative reverse-transcriptase PCR (qRT-PCR) was analyzed by using StepOnePlus Real-Time PCR System using SYBR-Green Mastermix (Applied Biosystems) and gene-specific primers as follows. For qRT-PCR, human GAPDH primers: sense 5′-GGGAAGCTTGTCATCAATGG-3′, antisense 5′-CATCGCCCACTTGATTTTG-3′; mouse β-actin primers: sense 5′-AGAGGGAAATCGTGCGTGAC-3′, antisense 5′-CAATAGTGATGACCTGGCCGT-3′ were synthesized from Invitrogen (Invitrogen, Carlsbad, CA); mouse PUMA primers (PPM4997A), human PUMA primers (PPH02204C), were purchased from Qiagen. RT-PCR was analyzed by using TITANIUM one-step RT-PCR kit (BD Biosciences, San Jose, CA) with the mouse NOS2 primers: sense 5′-GACAGCACAGAATGTTCCAG-3′, antisense 5′-TGGCCAGATGTTCCTCTATT-3′; mouse β-actin primers: sense 5′-GTGGGCCGCCCTAGGCACCAG-3′, antisense 5′-CTCTTTGATGTCACGCACGATTTC-3′.

### Western blot analysis

SDS-PAGE was conducted according to Towbin’s method as previously described (Du et al. 2009). Antibodies used were: NOS2 (BD Biosciences, San Jose, CA), PUMA, Lamin A/C, p21, IRF1, HDAC2 and H3AcK9 purchased from Cell Signaling Technology, Beverly, MA. The blots shown in the figures are representative of three experiments with similar results.

### Plasmid constructs and transient transfection assay

Human iNOS gene transfected-cell models were used to determine whether iNOS/NO regulated IRF1. The human iNOS expression plasmid was generated as described (Du et al. 2009). To determine if iNOS dose-dependently regulated IRF1 transcriptional activity, we established co-transfection assay. The consensus IRF1-luciferase reporter plasmid, pT109-IRF1 (3 × IRF1); consensus IRF1 oligonucleotides (5′-GAAAATGAAATT-3′) was cloned into the unique BamHI and XhoI site of luciferase reporter plasmid pT109, which contains 109 base-pairs of the herpes virus thymidine kinase promoter, driving expression of firefly luciferase, was confirmed by sequencing. In order to test whether IRF1 synergizes with p53 for target genes expression, a co-transfection was performed. Human TP53 and IRF1 expression plasmids, pCMV6-xl_5_-TP53 and pCMV6-xl_5_-hIRF1 were purchased from Origene, Rockville, MD. DNA transfections of cells were carried out in 6-well plates (Corning) by using Lipofectamine 2000 (Invitrogen) and MIRUS Trans-IT reagent (Mirus, Madison, WI) as previously described (Du et al. 2009).

### Adenovirus vectors and experimentally infection assay

For iNOS gene expression, adenovirus containing the human iNOS gene or control LacZ were infected in human hepatocytes and IRF1 protein levels determined. The University of Pittsburgh Pre-Clinical Vector Core Facility provided adenoviruses of the human iNOS (hiNOS) gene and its control, AdhiNOS and Adlacz.

### Assessment of NO induced HDAC2 activation

To test whether HDAC2 was involved in the mediation of iNOS/NO-induced IRF1 transcriptional activities and its target gene (PUMA and p21) expressions, NO donors were used. The expression of HDAC2, H3AcK9, IRF1, PUMA and p21 were measured.

The nuclear or total lysates from each treatment were prepared for western blot analyses. Cell lysate was resolved via SDS-PAGE and membranes were probed with the selected antibodies.

### Immunofluorescence staining and histopathology

The procedures were followed as described previously (Du et al. 2009). The primary antibodies: PUMA and IRF1 (Cell Signaling Technology), F-actin (Invitrogen), and iNOS (BD Biosciences) were purchased. Slides were viewed with Olympus Provis microscope, and FV1000 confocal microscope (Olympus). Formalin-fixed liver samples were embedded in paraffin, and stained with hematoxylin and eosin (H&E staining) for the assessment of inflammation and tissue damage.

### Nitric oxide production assessment

The Greiss assay was used as described previously (Du et al. 2009).

### ALT test

Serum alanine aminotransferase (ALT) levels were measured using the DRI-CHEM 4000 Chemistry Analyzer System (HESKA, Loveland, CO).

### TUNEL assay

The TUNEL assay was conducted following the manufacture introduction of In Situ Cell Death Detection Kit, Fluorescein, Roche. The liver tissue from I/R mice were subjected to TUNEL staining to detect apoptotic cells in Situ by labeling and detecting DNA strand breaks.

### Statistics

Data were processed using GraphPad Prism statistical software (version 6 or 8). Results were presented as mean ± standard deviation (SD). Experiments were carried out in duplicate or triplicate, and each was conducted a minimum of three times. For comparisons of functional performance between groups, an analysis of variance (ANOVA) or Student’s t test were applied. A *P*-value of *P* < 0.05 was considered significant.

## Results

### PUMA induction is dependent on iNOS in response to ischemia-reperfusion

**Fig. 1:**
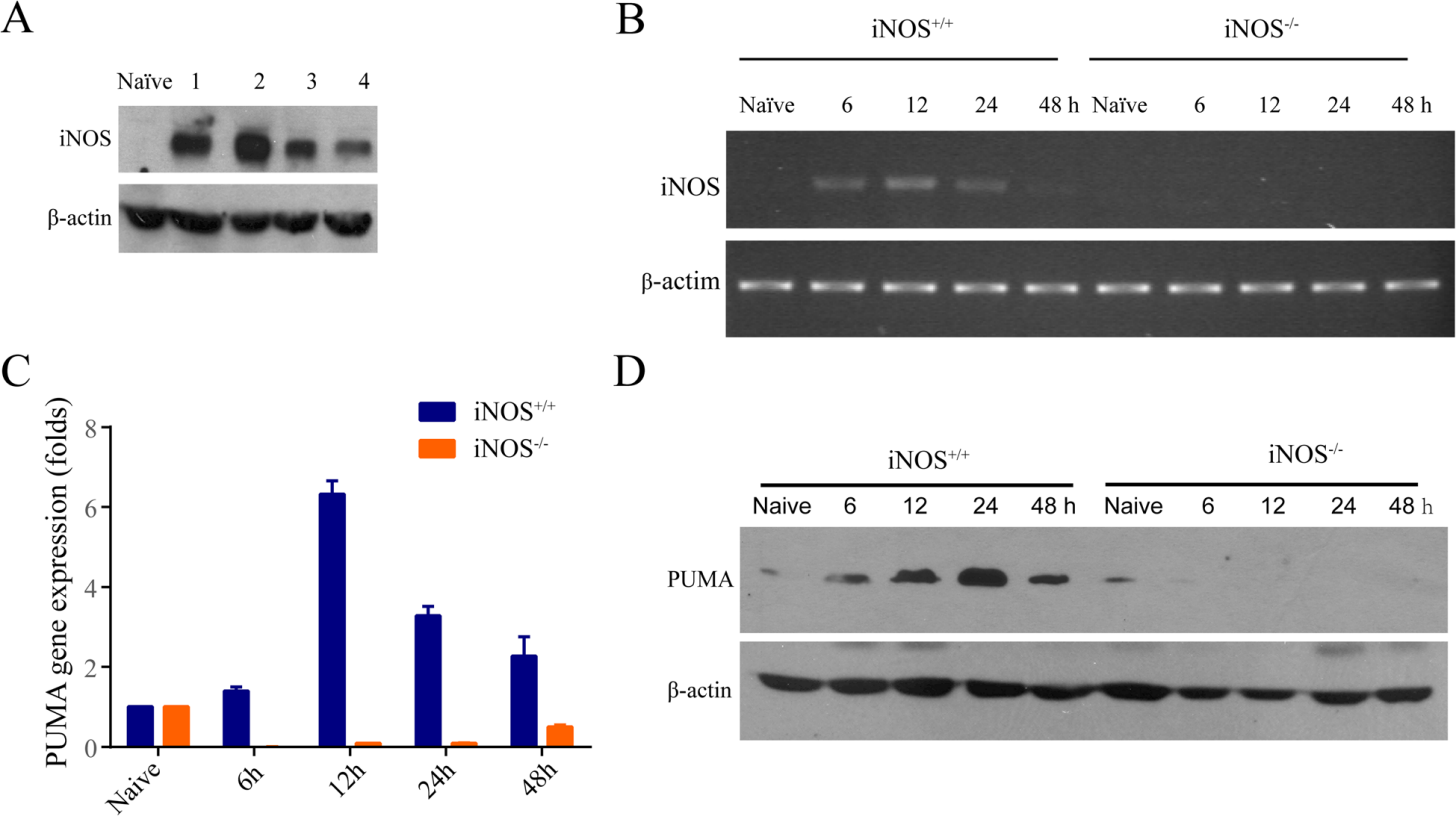
PUMA induction is dependent on iNOS wild-type in response to I/R. **a** Mouse liver I/R was performed with 1 h ischemia and 6 h reperfusion in C57BL/6 mice (*n* = 4). iNOS expression in the liver tissues was analyzed by Western blot. **b** Similar to (**a**), but 6, 12, 24 and 48 h reperfusion I/R were performed in iNOS^+/+^ and iNOS^−/−^ mice. RT-PCR detected mRNA coding for iNOS gene. **c** Liver tissues were collected from I/R iNOS^+/+^ and iNOS^−/−^ mice with ischemia for 1 h and reperfusion for the indicated times. qRT-PCR was carried out with primers for PUMA gene and normalized to β-actin in iNOS^+/+^ compared with iNOS^−/−^ mice at each time point, *P* < 0.0001. Data represent the mean ± the standard deviation (SD), *n* = 4. **d** Similar as (**c**), but PUMA expressions were analyzed in total proteins by Western blot

We and others previously described that I/R was known to induce cytokines and iNOS in liver (Ueki et al. 2010; Hamada et al. 2009; Tsung et al. 2006; Lee et al. 2001), as well as PUMA expression in intestine and heart (Wu et al. 2007; Toth et al. 2006). However, it is unknown whether PUMA induction is dependent on iNOS/NO in response to I/R. Mice (C57BL/6) were subjected to 60 min of partial warm liver I/R. Hepatic iNOS protein was strongly induced at 6 h after reperfusion in all mice, but not in normal (naïve) liver (Fig. 1a). Hepatic iNOS mRNA was induced in a time-dependent manner in iNOS^+/+^, but not in iNOS^−/−^ mice, with peak mRNA seen at 12 h after partial warm I/R (Fig. 1b). Next, we examined PUMA mRNA and protein expression after hepatic I/R injury. Surprisingly, hepatic I/R induced PUMA mRNA and protein expression in a time-dependent manner in iNOS^+/+^, but not in iNOS^−/−^ mice (Fig. 1c and d). These results indicate that PUMA induction is dependent on iNOS expression in liver I/R.

### iNOS/NO is required for IRF1 translocation to the nucleus and liver injury in ischemia-reperfusion mice

**Fig. 2:**
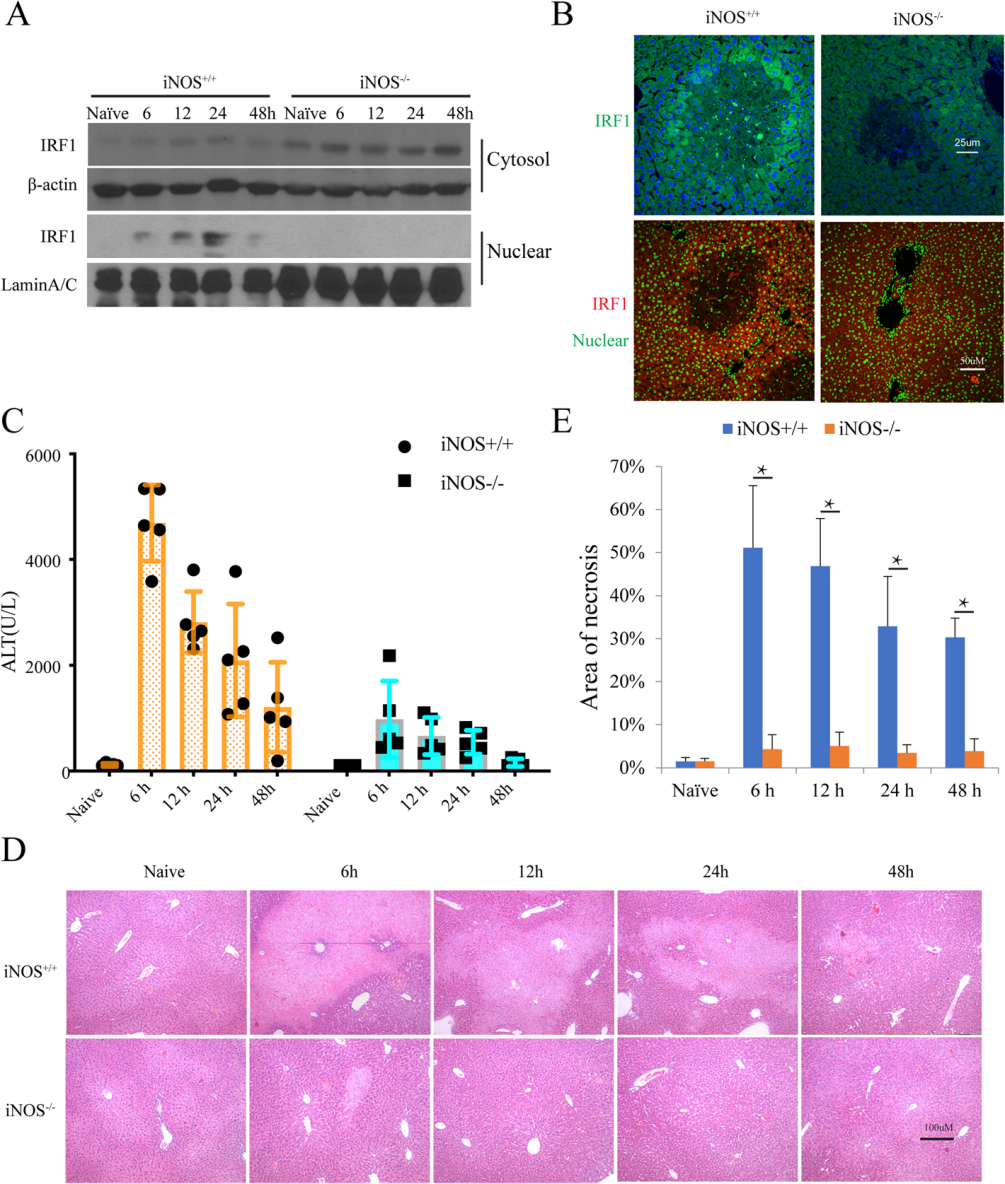
iNOS is required for IRF-1 translocation to the nucleus and liver injury in I/R mice. **a** iNOS^+/+^ and iNOS^−/−^ mice were used to generate I/R with 1 h ischemia and reperfusion as indicated. The nuclear and cytosolic proteins from the livers were analyzed by Western blot. **b** Livers from iNOS^+/+^ and iNOS^−/−^ I/R mice (6 h reperfusion) were subjected to immunofluorescence staining. Representative images are shown in the comparison of IRF1 expressions between the iNOS^+/+^ and iNOS^−/−^ mice. IRF1 is stained with FITC (green), and nucleus is stained with Hoechst dye (bis-benzimide) and is shown as blue color (upper). Moreover, to confirm the translocation of IRF1 to nucleus we used staining for IRF1 with Cy3 (red), and counterstaining for nucleus with SYTOX (green). Merging of the images shows the translocation to the nucleus of IRF1 as yellow color (lower). **c** ALT was detected in I/R iNOS^+/+^ vs. iNOS^−/−^ mice at the indicated time points. I/R more likely induced ALT releases with a time-course dependent manner in iNOS^+/+^ mice compared with that in iNOS^−/−^ mice, *P* < 0.0001. Data represent the mean ± SD, *n* = 5. **d** H&E staining of liver sections visualized in liver IRI in iNOS^+/+^ vs. iNOS^−/−^. Original magnification is × 100. **e** The necrotic areas were quantified with NIH ImageJ 2. Data are presented as mean ± standard deviation (*n* = 5, * *P* < 0.001 iNOS^+/+^ vs. iNOS^−/−^ at each time point)

In a previous study, we found that IRF1 plays an important functional role in mediating hepatic I/R injury in the liver transplant setting (Ueki et al. 2010). IRF1 also transcriptionally regulates iNOS (Kamijo et al. 1994; Matin et al. 1994) and PUMA (Gao et al. 2010). However, the role of iNOS/NO on IRF1 nuclear translocation and its transcriptional activity governing PUMA expression has not been studied. Hepatic I/R induced a time-dependent increase from 6 to 24 h in the expression of nuclear IRF1 protein which diminished by 48 h (Fig. 2a). In contrast, nuclear IRF1 protein was not detected in the iNOS^−/−^ mice. Cytosolic IRF protein was steady throughout the 6–48 h time course, and was not induced by I/R. Interestingly, naïve iNOS^−/−^ mouse livers exhibited slightly higher cytosolic IRF1 compared to the naïve iNOS^+/+^ controls (Fig. 2a). This difference might be due to the mice with iNOS deficiency increasing the basal level of IRF1 or possible stabilization of IRF1 cytosolic protein in the absence of induced-NO synthesis. Confocal microscopy confirmed these findings with strong IRF1 nuclear staining seen around the areas of tissue damage in the iNOS^+/+^ liver after I/R, but not in the iNOS^−/−^ mice (Fig. 2b upper). To further verify translocation of IRF1 to the nucleus we used staining for IRF1 (Red) and counterstaining for nucleus (Green). Merging the images (Yellow) demonstrates the nuclear localization of IRF1 in the liver of iNOS^+/+^ mice after I/R, compared with that of iNOS^−/−^ mice (Fig. 2b lower). These results suggest that iNOS expression is required for I/R-induced IRF1 nuclear translocation. To test whether iNOS/NO induced IRF1 contributes to liver IRI, we measured serum ALT levels. As expected iNOS^−/−^ mice exhibited markedly lower liver IRI compared to iNOS^+/+^ indicated by ALT levels (Fig. 2c). Moreover, liver histology showed the severe cell death in iNOS^+/+^ mice compared with the iNOS^−/−^ mice with peak necrosis seen at 6–12 h, and decreasing by 48 h (Fig. 2d, e). These findings broadly extended our previous results that IRF1 is an effector of IRI (Ueki et al. 2010) which is tightly controlled by iNOS.

### iNOS/NO mediates IRF1 transcriptional activity through a positive feedback mechanism

**Fig. 3:**
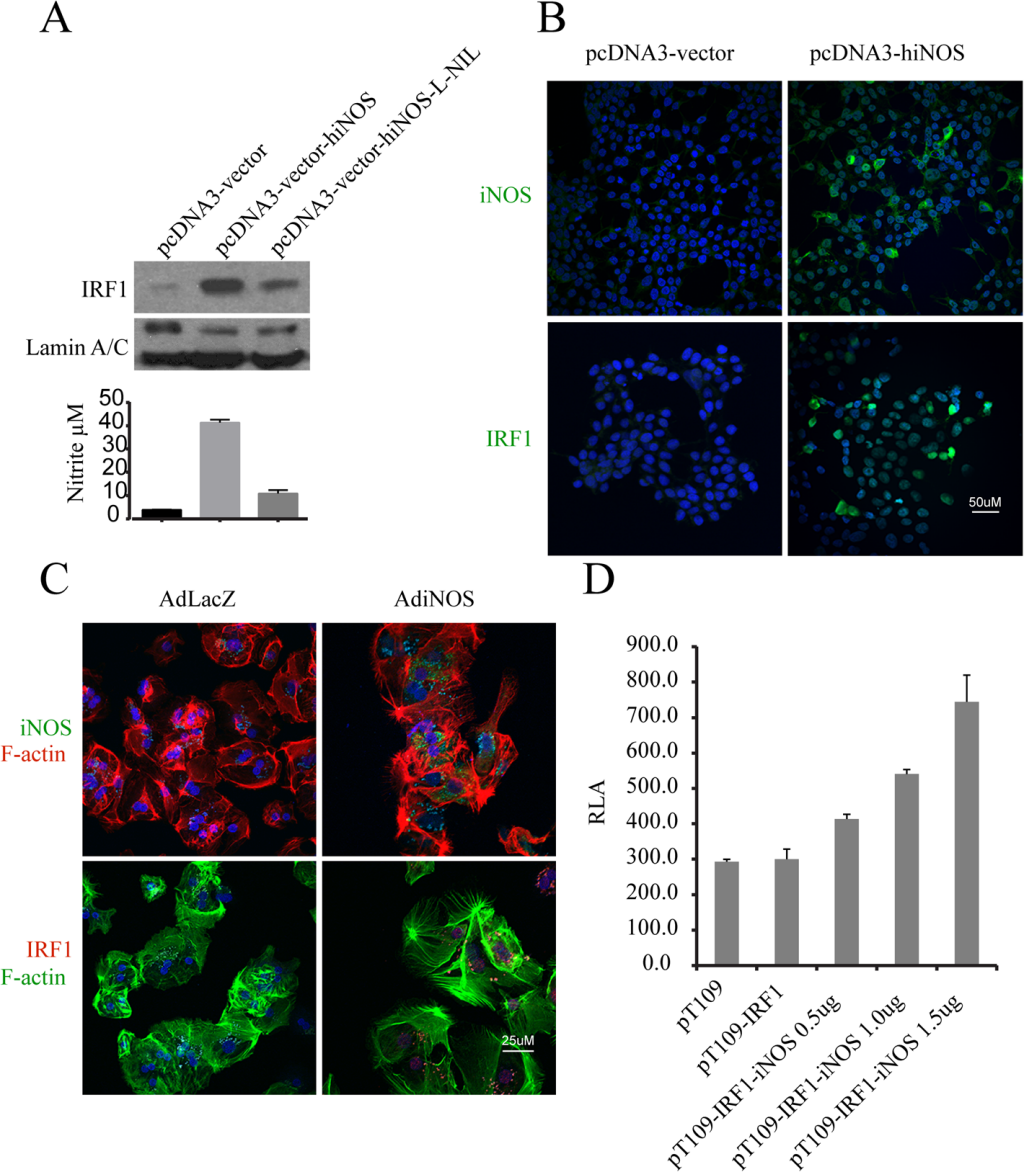
iNOS regulation of IRF1 transcriptional activity. **a** 293 T cells were transfected with plasmids pcDNA3 or pcDNA3-hiNOS for 24 h, and treated with L-NIL (100 μM, 24 h). The iNOS/NO-induced nuclear IRF1 was analyzed by Western blot (upper). Similar as (upper), but the NO production was detected (lower). **b** Similar to (**a**) upper, but the iNOS/NO induced-IRF1 was evaluated by immunofluorescence staining, green: iNOS or IRF1. **c** Human hepatocytes were infected by AdhiNOS or Adlacz for 24 h. Representative images of immunofluorescence staining are shown for IRF1 expression, green: iNOS; red: IRF1. **d** 293 T cells were transfected with pT109-IRF1 (0.5 μg) and iNOS expression plasmid, pT109-IRF1-iNOS with different concentrations of iNOS. Total amounts of plasmid DNA were kept constant by adding the empty pcDNA3A vector. Transcriptional activities of IRF1 were analyzed by luciferase assay (RLA: relative luciferase activity), cells transfected with pT109-IRF1-iNOS vs. pT109-IRF1, *P = 0.0003. The data shown are representative of three experiments with similar results

Given iNOS deficiency abrogated IRF1 induction as a transcription factor in hepatic I/R, we next sought to determine if iNOS over-expression increased IRF1 nuclear translocation. Over-expression of hiNOS was performed by transfecting pcDNA3-hiNOS plasmid into 293 T cells. Over-expression of hiNOS induced NO synthesis as expected measured by nitrite, and was attenuated by iNOS inhibitor, L-NIL (Fig. 3a). Overexpression of hiNOS also markedly induced IRF1 nuclear protein, which was partially reversed by L-NIL (Fig. 3a). Likewise, IRF1 nuclear localization was triggered by over-expression of iNOS compared to the control pcDNA3 vector (Fig. 3b). Similar to the 293 T cells, the increased nuclear localization of IRF1 was also observed in human hepatocytes infected with AdhiNOS compared to the control AdlacZ (Fig. 3c). IRF1 nuclear translocation is a prerequisite for IRF1 to act as a transcription factor. To further explore IRF1 transcriptional activation, we used an IRF1 reporter assay. Our IRF1 reporter plasmid (pT109-IRF1) carries 3 copies of the IRF1 response-element and was co-transfected with iNOS expression plasmid in 293 T cells. Over-expressions of hiNOS dose-dependently increased human IRF1 transcriptional activity (Fig. 3d) and is consistent with the notion that iNOS/NO drives IRF1 nuclear localization and transcriptional activity. Together, these findings indicate a signaling axis of iNOS/NO-IRF1-PUMA in hepatocytes. Since cytokine-induced IRF1 activates transcription of the iNOS gene (Kamijo et al. 1994; Martin et al. 1994), our findings are consistent with a positive-feedback mechanism where cytokine-induced IRF1 transcriptionally activates the iNOS gene, and iNOS-mediated NO synthesis triggers IRF1 gene expression and IRF1 nuclear translocation.

### iNOS inhibition reduces IRF1 signaling, PUMA expression, and liver IRI

**Fig. 4:**
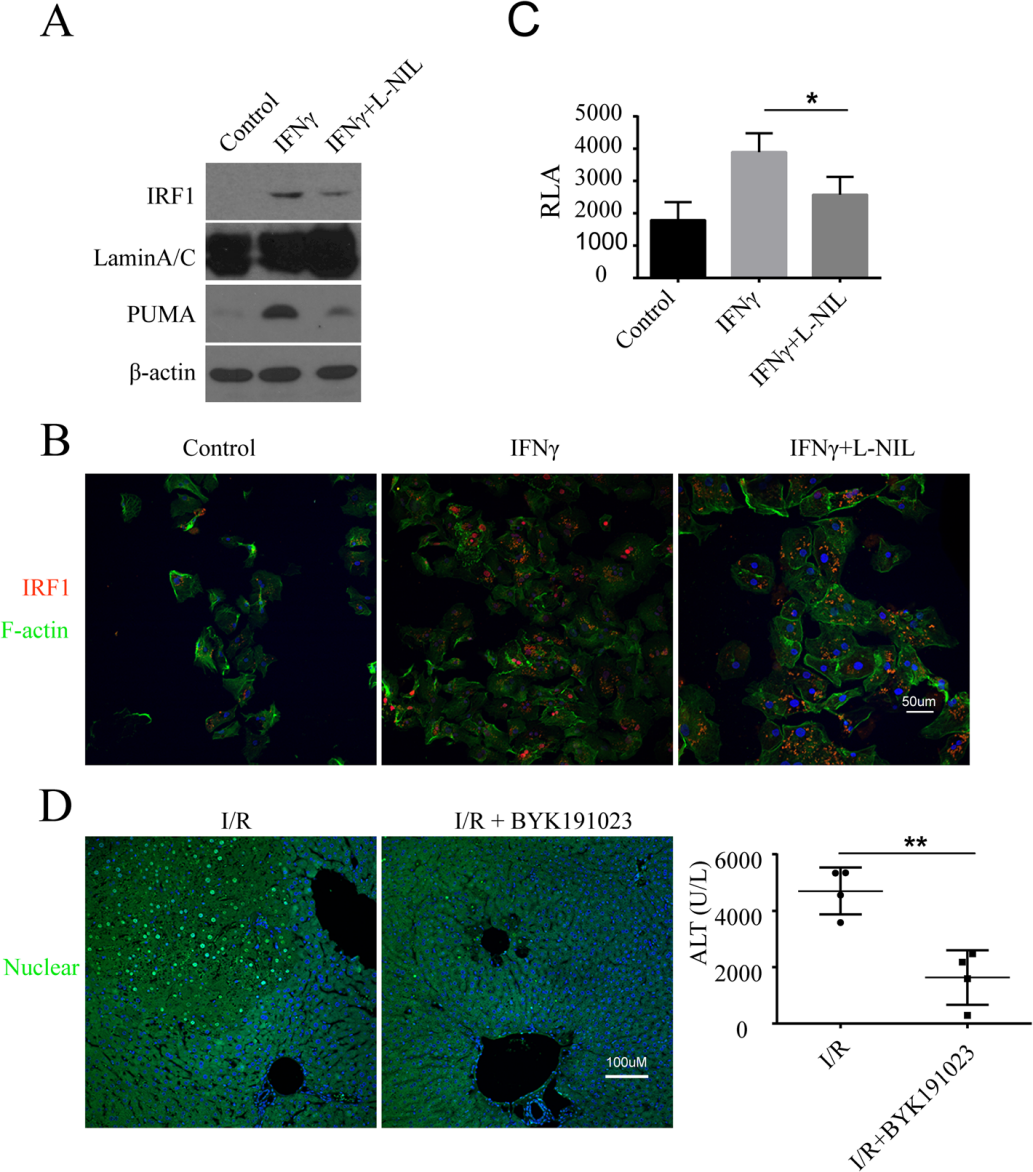
iNOS inhibition reversed the iNOS/NO induced signaling. **a** Human hepatocytes were treated with L-NIL (100 μM, 24 h), and IRF1 (nuclear extracts) and PUMA (whole cell extracts) were analyzed by Western blot. **b** Similar to (**a**), but representative images of immunofluorescence staining are shown, red: IRF1; green: F-actin; blue: nucleus. **c** Mouse hepatocytes were transfected with IRF1-luciferase reporter for 24 h, and followed the treatments as indicated for 9 h. Luciferase reporter assay was performed. L-NIL decreased IFNγ-induced IRF1 transcriptional response, *P = 0.03. The data shown are representative of three experiments with similar results. **d** Warm I/R mice (*n* = 4) were used for the study of iNOS inhibition reducing liver injury. Ischemia was performed for 1 h, and then reperfusion with the treatment of BYK191023 (60 mg/kg, 6 h). TUNEL staining of apoptotic cells with green color on the liver tissues (left) and ALT concentrations (right) were reduced by BYK191023 vs. the controls, **P = 0.003

To further verify that iNOS up-regulates IRF1 expression and its downstream transcriptional activity, primary human hepatocytes were treated with IFNγ with/without iNOS inhibitor L-NIL. As expected, IFNγ increased IRF1 and PUMA protein expression (Fig. 4a) and IRF1 staining in living cells (Fig. 4b). Noteworthy, induction of both IRF1 and its target PUMA was abrogated by iNOS inhibition with L-NIL (Fig. 4a and b). Similarly, iNOS inhibition with L-NIL also decreased IFNγ-induced IRF1 transcriptional activity in the IRF1-luciferase reporter assay (Fig. 4c). Finally, to determine the impact of iNOS on apoptotic cell death in vivo after I/R, mouse livers were examined by TUNEL staining. The increased TUNEL staining was observed 6 h after warm I/R, and this was diminished by the iNOS inhibitor, BYK191023 (Fig. 4d). Likewise, liver damage was also improved by BYK191023 with decreased serum ALT levels (Fig. 4d).

### IFNγ-induced PUMA is dependent on p53 and IRF1 synergistically targets PUMA expression with p53

**Fig. 5:**
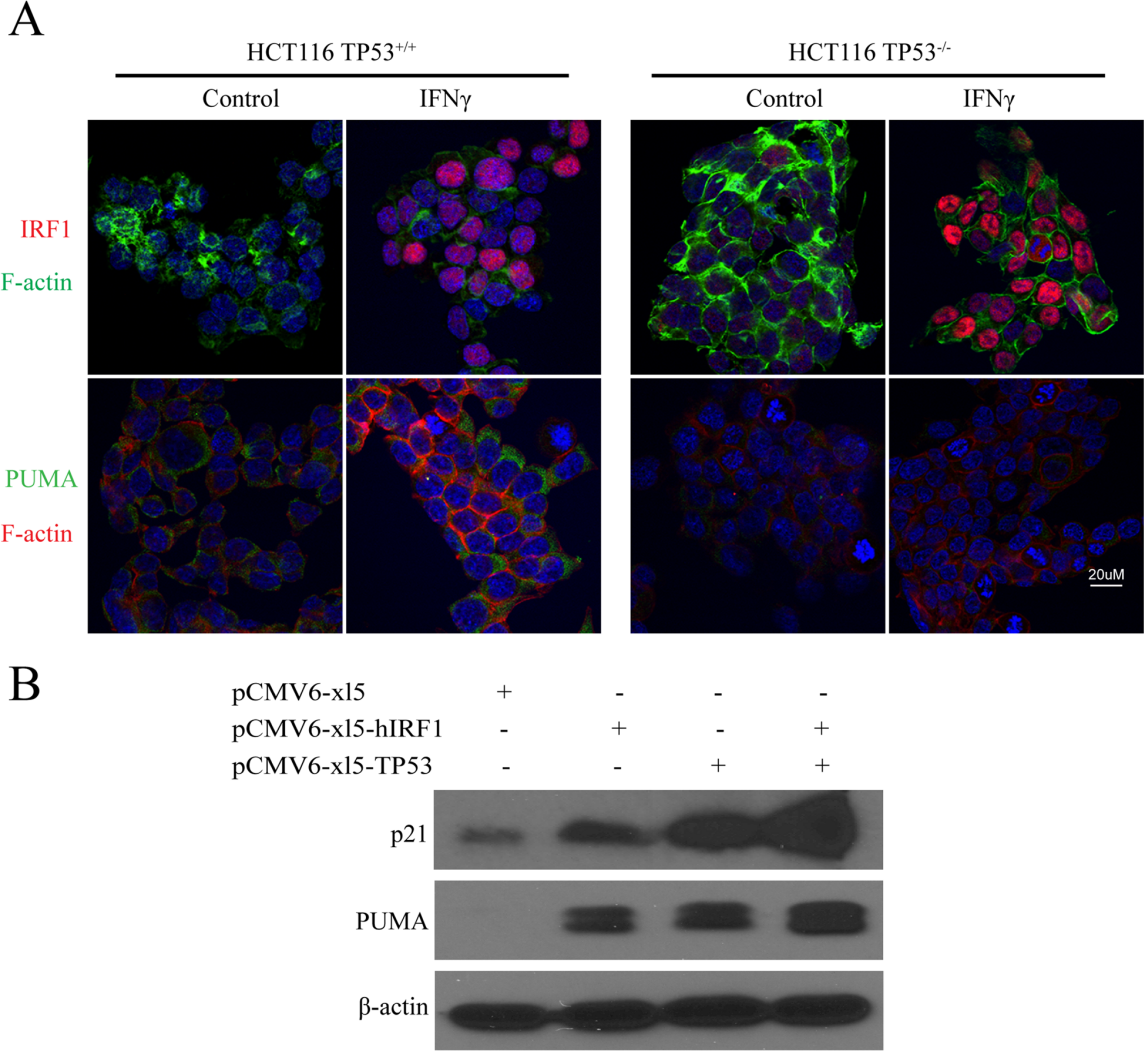
IRF-1 synergistically targets PUMA gene expression with p53. **a** HCT116TP53^+/+^ and HCT116TP53^−/−^ cells were treated with IFNγ (24 h). Immunofluorescence staining was performed with the indicated antibodies. Representative images are shown, red: IRF1; green: PUMA. **b** 293 T cells were transfected with pCMV6-xl_5_-hIRF1 (3 μg) or pCMV-xl_5_-TP53 (1 μg); and co-transfected with pCMV6-xl_5_-hIRF1 (3 μg) and pCMV-xl_5_-TP53 (1 μg) for 24 h. Total proteins were analyzed by Western blot with the indicated antibodies

Previous studies indicated that NO positively mediates p53 signaling (Forrester et al. 1996; Hemish et al. 2003), and NO regulates some gene expression including PUMA, which is dependent on p53 wild-type expression (Li et al. 2004). Since cytokines induce iNOS and IRF1, we tested whether cytokine-induced PUMA is dependent on p53, and whether activated IRF1 synergizes with p53 for the transcription of PUMA and p21. HCT116TP53^+/+^ (p53 wild-type) and HCT116TP53^−/−^ (p53 knockout) human colon cancer cell lines were used to further examine IFNγ induction of IRF1 and PUMA. IFNγ markedly induced IRF1 and PUMA protein expression in the HCT116TP53^+/+^ cells (Fig. 5a). Surprisingly, IRF1 was induced in the HCT116TP53^−/−^ cells, but PUMA expression was not detected with p53 deficiency (Fig. 5a). Hence, IFNγ-induced PUMA expression was p53-dependent, while IFNγ-induced IRF1 expression was p53-independent. Furthermore, although PUMA gene is transcriptionally regulated by p53 (Yu et al. 2001; Nakano et al. 2001; Yu and Zhang 2003) or IRF1 (Gao et al. 2010), it is unknown if IRF1 can synergize with p53 for PUMA expression. A co-transfection experiment was conducted to overexpress IRF1 and p53 in 293 T cells, and observe for synergistic effects on the induction of PUMA. We also examined for effects on p21, since it is also a target gene of IRF1 and p53 (Tanaka et al. 1996). Overexpression of either IRF1 or p53 increased both PUMA and p21 protein expression, while co-transfection of IRF1 and p53 together produced additive or synergistic effects on the induction of PUMA and p21 (Fig. 5b). Given that iNOS expression and induced NO synthesis up-regulates p53 (Forrester et al. 1996; Hemish et al. 2003) and IRF1, and cytokine-induced PUMA expression is dependent on wild-type p53, our findings are consistent with signaling pathways where iNOS/NO regulate IRF1 and p53 synergistically to transcriptionally activate PUMA (and possibly other target gene expression).

### iNOS/NO-induced HDAC2 activity up-regulates IRF1 transcription and nuclear localization

**Fig. 6:**
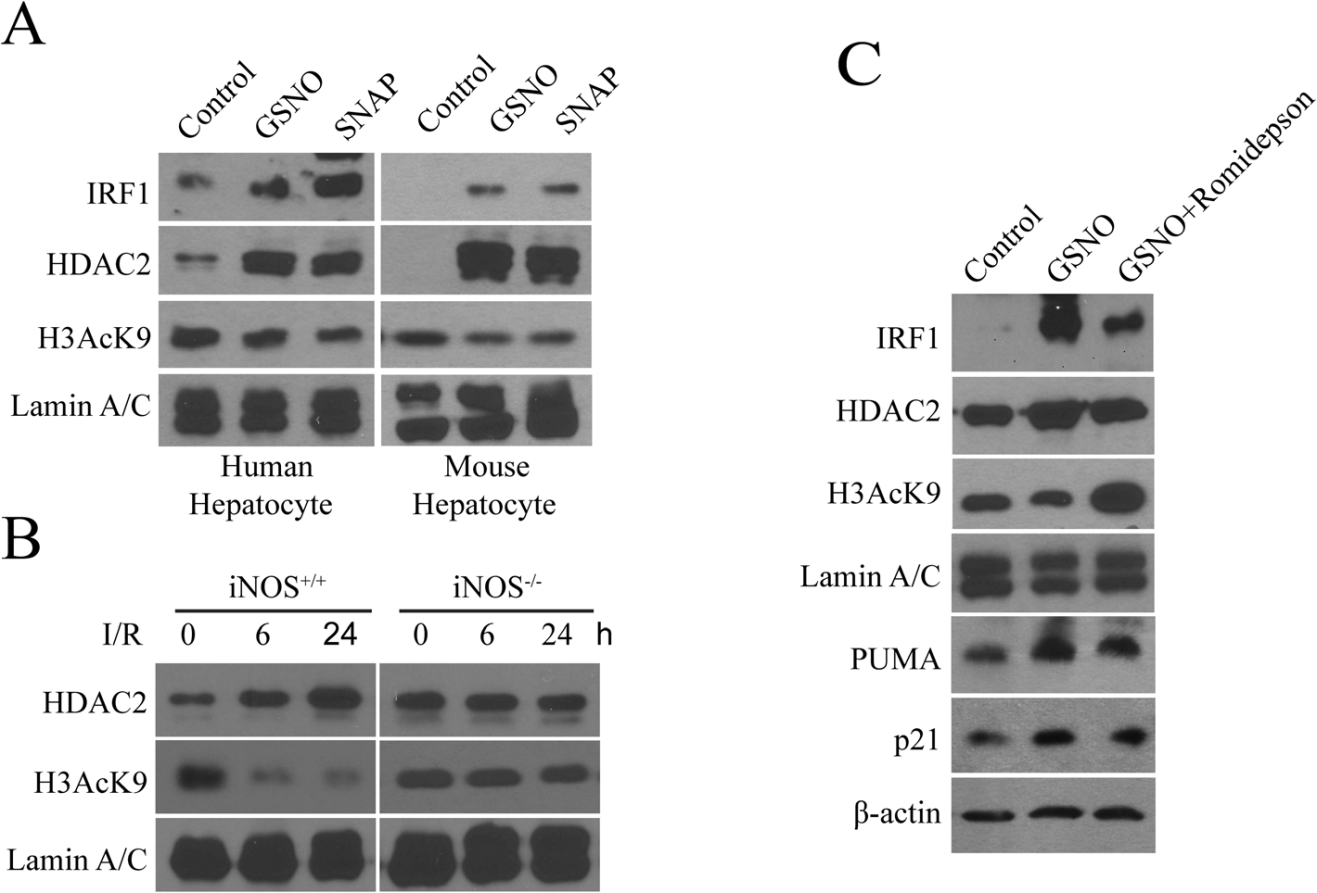
iNOS/NO was required for HDAC2 activity which up-regulated IRF1 nuclear translocation. **a** Mouse (left) and human (right) hepatocytes were treated with GSNO (1 μM) or SNAP (500 μM) for 3 h, respectively. Nuclear expressions of IRF1, HDAC2 and H3AcK9 were analyzed by Western blot; lamin A/C was loading controls. **b** Mouse hepatic I/R were performed 1 h ischemia and a various times of reperfusion as indicated. The nuclear expressions of HDAC2 and H3AcK9 in liver tissues were measured by Western blot. **c** 293 T cells were treated with GSNO (1 μM) and romidepsin (5 μM) for 3 h. The expressions of IRF1, HDAC2 and H3AcK9 (nuclear extracts), and PUMA and p21 (whole cell extracts) were analyzed by Western blot

Since histone deacetylases (HDACs) have been shown to modulate certain gene expression (Seto and Yoshida 2014), we tested whether iNOS/NO up-regulated IRF1 via HDAC activation. Mouse and human hepatocytes were treated with NO donors, GSNO or SNAP. Western blot analyses of nuclear proteins showed that NO donors increased IRF1 nuclear protein levels, and HDAC2 expression in primary human and mouse hepatocytes (Fig. 6a). Noteworthy, the NO donors also decreased histone H3 acetylation at lysine 9 (H3AcK9) expression (Fig. 6a), suggesting that H3AcK9 is a substrate of HDAC2. These results indicate that NO-activated HDAC2 regulates the acetylation state of chromatin in hepatocytes.

**Fig. 7:**
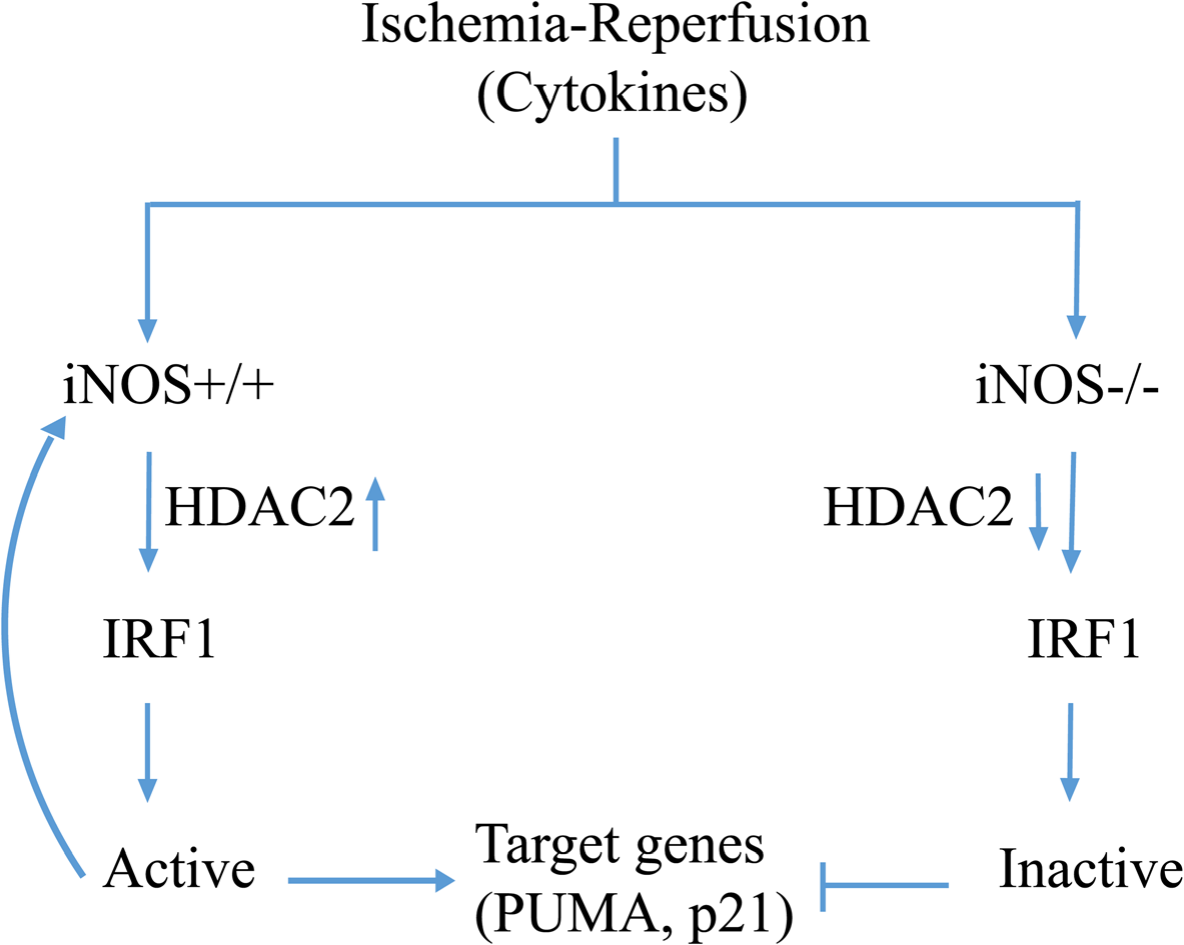
Schematic of the proposed model of iNOS/NO-mediated IRF1 activation in response to hepatic I/R in mice. Ischemia and reperfusion induces iNOS/NO, which activates IRF1 transcriptional activities. This process requires iNOS/NO induced HDAC2 activation to catalyze deacetylation of histone H3. On the other hand, iNOS gene deficiency decreases IRF1 and HDAC2 activities. The activated IRF1 as a transcription factor is translocated into the nucleus, where it regulates transcription of the target genes associated with cell death and cell cycle repression such as, iNOS, PUMA and p21. A positive feedback loop between IRF1 and iNOS may lead to IRF1 continuatively activated. Inhibition of HDAC2 by its inhibitor leads to an increase in histone H3 acetylation, and a decrease in IRF1 nuclear translocation and its target gene expressions (see Results and Discussion). I/R induced IRF1 activation requires iNOS/NO, which recruits HDAC2 as a co-activator to mediate chromatin modification

To further investigate if iNOS/NO was required for the HDAC2 expression and enzyme activity in vivo, we used hepatic I/R in iNOS^+/+^ and iNOS^−/−^ mice. I/R induced a time-dependent increase in the HDAC2 expression, and a decrease in the H3AcK9 expression in the iNOS^+/+^ mice, but not in iNOS^−/−^ mice (Fig. 6b). These results indicate that I/R increases HDAC2 and decreases H3AcK9 expression in an iNOS-dependent manner. To test the effect of NO-dependent HDAC2 on the nuclear expression of IRF1 and the expression of its target genes, PUMA and p21, 293 T cells were stimulated by NO donor, GSNO, with/without romidepsin, an inhibitor of HDAC1/2. The nuclear proteins were subjected to immunoblotting analysis of IRF1, HDAC2 and H3AcK9 expression, and total proteins for the analysis of PUMA and p21. GSNO induced IRF1, PUMA, and p21 expression, which were decreased by romidepsin (Fig. 6c). This result confirmed that NO induced HDAC2 is involved in the regulation of IRF1 translocation to the nucleus and its target gene transcriptional expression. In contrast, the GSNO decreased H3AcK9 expression which was markedly increased in the presence of romidepsin. These findings indicate that iNOS/NO up-regulates IRF1 translocation to the nucleus, and transcriptional activity of certain target genes is at least partially dependent on NO-mediated histone acetylation status. Collectively, our data supports an important mechanism involved in iNOS/NO-IRF1-PUMA signaling axis through HDAC2 activation in response to liver I/R (Fig. 7).

## Discussion

In this study, we demonstrate a new mechanism of iNOS/NO regulating the IRF1 signaling pathway. Hepatic IRF1 and PUMA expression is induced in an iNOS-dependent manner in response to warm liver I/R. The induction of iNOS increases, while genetic deficiency or biochemical inhibition of iNOS decreases IRF1 transcriptional activity. iNOS/NO up-regulates IRF1 signaling via a positive-feedback loop. IRF1 activated-PUMA expression is dependent on p53 wild-type, and synergistically up-regulated by IRF1 and p53. Moreover, iNOS/NO up-regulates IRF1 and its target gene expression of PUMA, as well as p21 by increasing HDAC2 and decreasing H3Ack9 expressions in vitro in hepatocytes, and in vivo in warm liver I/R. These findings provide the novel mechanistic insights into how iNOS/NO signals mediates IRF1 and PUMA signaling in response to I/R (Fig. 7).

It is well documented that iNOS plays a key role in I/R injury. In a pig liver transplantation study, iNOS expression in Kupffer cells and neutrophils triggered hepatic I/R injury (Zhai et al. 2013; Isobe et al. 1999). In a warm IR injury study, IL-6 was increased at 6 h and reduced at 24 h; while TNFα and IFNγ were continually increased from 3 to 24 h (Hamada et al. 2009). Interestingly, decreased induction of IL-6 and IFNγ was observed in iNOS^−/−^ mice compared with iNOS^+/+^ mice (Hamada et al. 2009). Clearly, iNOS/NO is a critical player affecting cytokine production through an autocrine or/and paracrine mechanism in response to hepatic I/R.

Our study provides evidence that iNOS/NO triggers hepatocyte death through up-regulation of the IRF1-PUMA signaling axis, which further contributes to liver I/R injury. NO-stress in the cellular microenvironment can affect some transcription factors to upregulate PUMA gene expression. NO upregulates p53 (Forrester et al. 1996; Hemish et al. 2003) and promotes phosphorylation at serine 15, which transcriptionally upregulates its target genes (Brüne 2003). Although PUMA is an essential mediator of p53-dependent and p53–independent apoptotic pathways (Jeffers et al. 2003), NO treatment induces PUMA expression dependent on p53 (Li et al. 2004). Another study has shown that p53 and iNOS form a negative-feedback circuit, in which p53 down-regulates iNOS (Forrester et al. 1996; Hemish et al. 2003). NO activates FOXO1 entering the nucleus, and up-regulates PUMA gene when SIRT1 is negatively targeted (Hughes et al. 2011). In response to cytokines or growth factor withdrawal, PUMA, together with Bim, functions as FOXO3a downstream target to mediate a stress response when Myc and PI3K/Akt signaling is down-regulated (You et al. 2006). NF-κB (Wu et al. 2007) or IRF1 (Gao et al. 2010) transcriptionally regulates PUMA by directly binding to its response-element(s) in the promoter. Interestingly, our study reveals that IRF1 translocation to the nucleus and PUMA expression was found in iNOS wild-type mice compared with that in iNOS knockout mice in response to I/R (Figs. 1 and 2). Since iNOS is a target gene of IRF1 (Kamijo et al. 1994; Martin et al. 1994), our results suggest that there is a positive feedback mechanism, in which I/R-mediated IRF1 activation is further enhanced by iNOS/NO. Together, our data indicate complex signaling where I/R-induced iNOS/NO induces IRF1 and PUMA expression, leading to IRF1-PUMA mediated hepatocyte death and liver injury.

As described above, iNOS/NO is required for the upregulation of IRF1. Cytokine-induced IRF1 is independent of p53, but induced PUMA expression is dependent of p53 wild-type. Moreover, our previous study indicates that NO also up-regulates p53 (Forrester et al. 1996). Therefore, we may infer that iNOS/NO induced IRF1 and p53 synergistically work to transcriptionally mediate their target gene expressions, such as PUMA and p21 under certain conditions. As p21 is an important player in cell cycle arrest, iNOS/NO-induced IRF1 and p53 pathway may additionally mediate p21-induced inhibition of cell cycle in liver I/R. The important relationship between IRF1 and p53 is illustrated in the case of p53 deficiency or mutation, where IRF1 can function independently, but in the presence of wild-type p53, the two may act synergistically. Hence, a cooperative mechanism between IRF1 and p53 exists in the IRF1-PUMA or IRF1-p21 pathway for the regulation of cell fate.

It is known that some interferon-stimulated genes require protein deacetylase activity, such as HDAC (Nusinzon and Horvath 2003; Chang et al. 2004; Marie et al. 2018). Moreover, some IFN-stimulated genes are inhibited by trichostatin A (TSA) or romidepsin (Nusinzon and Horvath 2003; Chang et al. 2004; Marie et al. 2018). Interestingly, HDACs augmenting cytokine-induced iNOS gene expression has been reported (Yu et al. 2002). Overexpression of HDAC2 (deacetylation) enhanced, but TSA (hyperacetylation) inhibited cytokine induction of both iNOS and the NF-κB element promoter (Yu et al. 2002). In the current study we found that iNOS/NO was able to induce HDAC2 activity (promoted histone H3 deacetylation), which up-regulated IRF1 and PUMA expression in in vivo and in vitro. Therefore, iNOS/NO-induced HDAC enhanced IRF1-PUMA-induced cell death capacity due to hyperactivation of the IRF1 target-gene PUMA.

The consequence of NO-induced histone deacetylation may play an important role in regulating iNOS-dependent genes (e.g. IRF1) in response to I/R. Our results indicate that NO-induced HDAC2, by activating the expression of IRF1 and PUMA, regulate cell death. Thus, HDAC2 may be a relevant target for HDAC inhibitors to prevent I/R injury. On the other hand, iNOS/NO is regarded as a principal mediator of NO-dependent S-nitrosylation. A large part of NO-dependent gene transcription in mammalian cells is conferred by tightly regulated and specific protein S-nitrosylation, through either direct modification of transcriptional regulators or upstream intermediates (e.g. HDACs) in the respective signaling pathways (Datta et al. 2013; Isobe et al. 1999; Sha and Marshall 2012).

PUMA is a critical player not only to mediate apoptosis (Yu and Zhang 2008), but also to regulate acetaminophen-induced necrosis and liver damage (Chen et al. 2019). PUMA has also been documented to amplify necroptosis signaling by activating cytosolic DNA sensors involved in TNF-driven necroptotic death (Chen et al. 2018). Given the regulatory role of PUMA in cell death, several studies have reported the use of PUMA inhibitors to reduce cell death (Chen et al. 2019).

## Conclusion

This study provides novel insights into the mechanism of iNOS/NO regulating IRF1-PUMA signaling, which may play an important regulatory role in liver I/R and other inflammatory responses and tissue injury. Understanding the cross-talk between iNOS/NO and IRF1-PUMA pathway in I/R may represent a therapeutic target for hepatic injury.

## Data Availability

Not applicable.

## Abbreviations

ALT: Serum alanine aminotransferase
HDAC: Histone deacetylase
]IFNγ: Interferon-γ
iNOS: Inducible nitric oxide synthase, also known as NOS2
I/R: Ischemia-reperfusion
IRI: Ischemia-reperfusion injury
IRF1: Interferon regulatory factor-1
p21: p21^CIP1/WAF1^ protein
PUMA: p53 up-regulated modulator of apoptosis
TNFα: Tumor necrosis factor-α
